# Impact of legislative reform on coercive measure use in child and adolescent psychiatry: A controlled interrupted time series analysis

**DOI:** 10.1007/s00787-026-03035-1

**Published:** 2026-05-18

**Authors:** Astrid Moell, Erik Pettersson, Riittakerttu Kaltiala, Niklas Långström

**Affiliations:** 1 Centre for Psychiatry Research, Department of Clinical Neuroscience, Karolinska Institutet, & Stockholm Health Care Services, Region Stockholm, Norra Stationsgatan 69, 7th floor, 113 64 Stockholm, Sweden; 2 Department of Medical Epidemiology and Biostatistics, Karolinska Institutet, Stockholm, Sweden; 3 Faculty of Medicine and Health Technology, Tampere University, Tampere, Finland; 4 Department of Adolescent Psychiatry, Tampere University Hospital, Tampere, Finland; 5 Vanha Vaasa Hospital, Vaasa, Finland

**Keywords:** Coercive measures, Restrictive interventions, Inpatient care, Legislative reform

## Abstract

Evidence on legislative reforms to reduce coercive measures remains limited. We evaluated short-to-medium-term effects of Sweden’s July 2020 stricter legal regulation of coercive measures in youth < 18 years. In a controlled interrupted time-series study, we used 2017–2021 registry and electronic medical record data from inpatient child and adolescent mental health services (CAMHS) in Region Stockholm, Sweden. Controls included Finnish CAMHS and Region Stockholm young adult psychiatric patients (18–24 years). Monthly outcomes were rates of seclusion, mechanical restraint, involuntary medication and a composite measure, mean durations, and sedative medication use. Segmented regression estimated level- and trend changes, adjusting for pre-intervention trends. Among 7,075 admissions (3,840 individuals) to Stockholm inpatient CAMHS, involuntary medication *increased* immediately in minors relative to young adults (incidence rate ratio 2.49, 95% CI 1.63–3.81), with no other changes in patient-day-standardised models. Comparisons with Finnish CAHMS provided no consistent evidence of reduced coercion. Mean durations also increased in minors versus young adults (12% for seclusion; 6% for mechanical restraint), while sedative medication use remained stable. Findings were robust in sensitivity analyses. Contrary to the reform’s aims, stricter legislation alone was not associated with reduced coercive measure use.

**Supplementary Information:**

The online version contains supplementary material available at https://doi.org/10.1007/s00787-026-03035-1.

## Introduction

Coercive measures remain widely used in inpatient child and adolescent mental health services (CAMHS), despite ethical, clinical, and legal concerns. In a recent systematic review, a median of 17.5% of patients admitted to inpatient CAMHS were subjected to at least one coercive measure [1]. Stricter legislation aimed at reducing coercive measure use has been introduced in several countries, yet the impact of such reforms on clinical practice remains uncertain.

Most available evidence comes from adult psychiatry, where legislative restrictions suggest mixed results. Some studies report reductions in specific coercive measures accompanied by concurrent increases in other coercive measures [2–4], whilst others report no overall change [5, 6]. Evidence from child and adolescent settings is scarce, with only two quantitative evaluations: one found no reduction in residential care for children with intellectual disability [7], whereas another reported substantial short-term reductions in Irish psychiatry, including CAMHS [8]. However, most existing studies rely on uncontrolled pre-post comparisons, unable to account for secular trends or concurrent system changes.

In Sweden, a legal reform effective from July 1, 2020, introduced stricter regulations on coercive measures for minors (< 18 years) in inpatient psychiatric care. Key amendments included shorter maximum durations per decision (mechanical restraint from four to one hour; seclusion from eight to two hours), stricter legal thresholds for use, and stronger necessity requirements. Seclusion is now limited to aggressive behaviour that seriously disrupts care for other patients, and mechanical restraint to the immediate prevention of serious harm to self; both permitted only when less restrictive measures are clearly insufficient (see Appendix pp2–3 for details on the legal framework and reform). Our previous qualitative study indicated that the implementation was challenging, as staff reported increased administrative burden, uncertainty in interpretation of the law, and a perceived shift towards sedative medication and seclusion rather than a reduction in overall coercion [9].

Since robust evidence on the effects of legislative reform on coercive practices remains limited, we conducted a controlled interrupted time-series (ITS) study examining whether the Swedish 2020 reform was associated with changes in monthly rates of coercive measures, their duration, and potential unintended increases in sedative medication use in Region Stockholm.

## Methods

### Study design

We conducted a quasi-experimental study using controlled ITS analyses to estimate the short-term and medium-term effects of the Swedish legislative change on the use of coercive measures in CAMHS. We used prospectively collected registry data from Region Stockholm and national Finnish registry data. The study protocol was preregistered on the Open Science Framework (https://doi.org/10.17605/OSF.IO/3ENY7). We followed Strengthening the Reporting of Observational Studies in Epidemiology (STROBE) guidelines [10].

### Participants and data

The study population consisted of one intervention series: CAMHS inpatients < 18 years treated in Region Stockholm; and two control series (a) young adult psychiatric inpatients aged 18–24 years treated in Region Stockholm, serving as a within-region control group not directly targeted by the legislative change; and (b) Finnish CAMHS inpatients aged < 18 years, serving as an external control group. We included all inpatient admissions within the age ranges with admissions or discharges during January 1, 2017–Dec 31, 2021. Further details are available in the Appendix (pp3–4).

We used data from Region Stockholm electronic medical records; timestamped data were available for admission, discharge, coercive measure use, and all inpatient medication administration. From Statistics Sweden registries, we obtained date of birth and legal sex, calculating age at admission and coercive measure exposure. Finnish data were obtained from the Care Register for Health Care for all CAMHS inpatient admissions during the study period; coercive measures were recorded per admission, but without exact timestamps. Population counts by single-year age groups were obtained from Statistics Finland (annual) and Statistics Sweden (monthly).

In the pre-registered protocol, we planned to use both a Swedish national dataset and the Region Stockholm dataset. However, after accessing the national dataset, we identified critical data-quality limitations precluding a valid ITS analysis: incomplete capture of coercive measures and coding changes over time, resulting in missing measures in some years. Therefore, all primary and secondary Swedish analyses used the Stockholm dataset. Region Stockholm is Sweden’s most populated region, with 24% of the nation’s children under age 18 residing there in 2021 (Statistics Sweden, open database).

### Interrupted time-series framework

We used segmented regression when analysing the ITS data, with controlled ITS as the primary framework. This involves adding concurrent comparison series (groups not directly targeted by the intervention), to help separate the intervention effect from secular trends and specific external events. Controlled ITS estimated immediate (level) and gradual (slope) intervention effects, while accounting for group-specific baseline trends and temporal patterns shared by intervention and comparison series [11].

The controlled ITS models were specified as:

$$\begin{array}{c}\:\mathrm{log}\left({\mu\:}_t\right)={\beta\:}_0+{\beta\:}_1{\mathrm{time}}_t+{\beta\:}_2{\mathrm{intervention}}_t\\+{\beta\:}_3{\mathrm{posttime}}_t+{\beta\:}_4\mathrm{group}+{\beta\:}_5({\mathrm{time}}_t\times\:\mathrm{group})\\+{\beta\:}_6({\mathrm{intervention}}_t\times\:\mathrm{group})+{\beta\:}_7({\mathrm{posttime}}_t\\\times\:\mathrm{group})+\mathrm{log}\left({\mathrm{offset}}_t\right)\end{array}$$

 where $\:{\mu\:}_{t}$denotes the expected outcome and $\:{\mathrm{offset}}_{t}$ the relevant denominator (e.g., patient-days or population at risk); $\:{\beta\:}_{6}$and $\:{\beta\:}_{7}$estimate differential level and slope changes versus controls.

Primary outcomes were analysed using both simple ITS (Stockholm CAMHS only) and controlled ITS. We conducted controlled comparisons for Stockholm CAMHS versus Stockholm young adults (within-region) and Finnish CAMHS (cross-national). No transition period was specified because the intervention date was well defined and immediate effects were plausible.

### Outcomes

Primary outcomes were monthly rates of patients subjected to a composite of three coercive measures (seclusion, mechanical restraint, and/or involuntary medication; in Finland also physical restraint) and to each of these three measures separately. Rates were based on monthly counts of exposed patients, standardised by patient-days (within-region Stockholm analyses) or population aged 0–17 years (Stockholm–Finland comparisons). In primary Stockholm analyses, patient-based rather than event-based rates were used to avoid disproportionate influence of patients with repeated or prolonged exposure. Further details are provided in the Appendix (p3).

Secondary outcomes were monthly mean duration of seclusion, mechanical restraint, or both combined; monthly proportions of patients receiving sedative medications, including antipsychotics, anxiolytics, hypnotics, and sedating antihistamines; and patient-day–standardised monthly rates of sedative doses. All sedative medications were analysed both as a composite and in clinically meaningful groups (see Appendix p4 for classifications).

### Exposure

The exposure was the legislative change regulating coercive measures in Swedish CAMHS (stricter requisites and shorter durations allowed), effective July 2020. It was coded as a binary indicator (0 before July 2020, 1 thereafter). We indexed time in months from series start, and post-intervention time as months since July 2020 (July = 0, August = 1, etc.), allowing estimation of immediate level changes and post-intervention trend changes.

Two potentially influential events close to the legal change were the incorporation of the UN Convention on the Rights of the Child into Swedish law on January 1, 2020, and the COVID-19 pandemic. Although Sweden had ratified the Convention in 1990, the 2020 change elevated it to statutory law; however, its practical implications for clinical practice remain unclear. Pandemic restrictions in Sweden required part-time or fully online schooling for post-secondary or high-school students (≥ 15 years) from March 2020 to March 2021. Finland was chosen as a comparison to help account for the impact of the pandemic on mental health services across the Nordic region. Finnish CAMHS experienced pandemic-related changes during the study period, including a substantial increase above predicted levels newly diagnosed psychiatric disorders [12].

### Data preparation

We aggregated all data into calendar months, though the unit of aggregation differed between datasets due to data structure. For the Stockholm data, patients subjected to coercive measures were counted in the month(s) the measure(s) occurred based on exact timestamps (a patient could contribute to multiple months if subjected to measures across months). In Finnish data, due to the absence of measure-specific timestamps, patients were counted only by admission month. Hence, each patient contributed at most one count per coercive measure type and admission (addressed in a sensitivity analysis, outlined below).

For the Stockholm data, we calculated monthly patient-days as the sum of all days between admission and discharge (included). To prevent overrepresentation, individuals contributed a maximum of one patient-day per calendar day, regardless of multiple admissions or discharges within a single day. Details on missing data are available in the Appendix (pp4).

### Statistical analysis

The effective sample size for ITS analyses was the number of monthly observations. Each series contributed 60 months (42 pre-intervention and 18 post-intervention). No formal power calculation was performed since all available data within the study period were included.

Count outcomes (patients subjected to coercive measures and sedative medication doses) were analysed using Poisson regression with a log link and log offset (monthly patient-days for within-Stockholm analyses; monthly population aged 0–17 for Finnish comparisons). Overdispersion was assessed using Pearson-based dispersion diagnostics; where the Poisson model indicated substantial overdispersion, we refitted using a negative binomial distribution. Effects were reported as incidence rate ratios for level and slope changes, obtained by exponentiating coefficients.

Due to Finnish data privacy restrictions precluding unified modelling, between-country comparisons were conducted using a two-stage approach [13]: country-specific interruption effect estimates and standard errors were obtained from separately fitted segmented regression models, and differences in coefficients on the log scale were then calculated assuming independence between datasets, and exponentiated to yield incidence rate ratios with 95% confidence intervals. Time-series plots for Stockholm-Finland comparisons are not presented, as population-standardised rates could be back-calculated to reveal counts below minimum reporting thresholds.

As an exploratory analysis, we examined event-based coercive measure rates standardised by patient-days in the Stockholm dataset. Monthly mean durations were log-transformed due to right skewness and analysed using linear segmented regression, with exponentiated coefficients interpreted as ratios (multiplicative changes) in monthly mean durations. Further, monthly proportions of patients receiving sedatives were analysed using logistic regression-derived odds ratios.

Residual diagnostics (autocorrelation and partial autocorrelation function [ACF/PACF] plots, and Ljung–Box tests; Durbin–Watson tests in addition for linear models) indicated varying degrees of autocorrelation. We applied Newey-West heteroskedasticity and autocorrelation consistent (HAC) standard errors with a lag of 12 months (pre-specified). Given indication of some residual autocorrelation, we conducted sensitivity analyses using an 18-month lag structure for primary outcomes in the Stockholm dataset (where coercive measures could be attributed to specific calendar months based on exact timestamps).

We conducted the following additional *sensitivity analyses* for the primary and secondary outcomes: (1) using a false intervention date (July 1, 2018) as a negative exposure control; (2) excluding eating disorder admissions to address potential distortion from prolonged admissions, increasing compulsory treatment rates, and changes in coercion registration during nasogastric feeding; (3) re-aggregating Stockholm data by admission month to mirror the Finnish registry structure; (4) applying multiple imputation for missing duration data; and (5) using geometric mean durations instead of log-transformed arithmetic means (see Appendix p4).

P-values < 0.05 were considered significant. We did not adjust for multiple comparisons across outcomes, as primary and secondary outcomes were pre-specified and represent clinically distinct constructs. However, the p-values for sedative medication subgroups should be interpreted cautiously, given multiple testing.

We used SAS (version 9.4) and R (version 4.5.1) for data cleaning, statistical analyses and creation of figures. The R package mice was used for multiple imputation [14].

### Lived experience commentary

The lived experience commentary was developed with three young adults with personal experience of coercive measures in inpatient CAMHS, recruited through national patient organisations and social media. After reviewing a plain-language study summary, they participated in a structured discussion with researchers about the findings and their implications. The commentary presented in this paper is based on these discussions.

## Results

In Region Stockholm, 25,568 psychiatric inpatient admissions involving 10,638 individuals were recorded between Jan 1, 2017, and Dec 31, 2021, including 3840 individuals aged < 18 years and 7200 aged 18–24 years (Table 1). In Finland 39,062 admissions of 18,104 individuals < 18 years were recorded over the same time period. Pre- and post-legislative periods were broadly similar regarding patient ages and length of stay; however, the proportion of female patients increased after the legal change, while the distribution of primary diagnoses varied over time. Cross-country comparison indicated major differences in diagnostic groups; substance use disorders where much more common in Stockholm compared to Finland, while the opposite was true for behavioural disorders.

**Table 1:** Characteristics of psychiatric inpatient admissions before and after Swedish July 2020 legal change, by age group and geographic location

Characteristic	Stockholm CAMHS (0–17 yrs)		Stockholm young adults (18–24 yrs)		Finnish CAMHS (0–17 yrs)	
	Pre	Post	Pre	Post	Pre	Post
	*n* = 4672; individuals = 2721	*n* = 2403; individuals = 1340	*n* = 12,638; individuals = 5237	*n* = 5855; individuals = 2615	*n* = 23,215; individuals = 12118	*n* = 15,847; individuals = 5986
Female sex	64.2% (2999)	73.4% (1763)	51.9% (6554)	55.4% (3246)	60.6% (14,395)	66.8% (10,224)
Age at admission, median (IQR), yrs	16 (15–17)	16 (14–17)	21 (19–23)	21 (19–23)	14 (12–16)	14 (12–16)
Length of stay, median (IQR), days	2 (2–6)	2 (1–5)	3 (2–7)	3 (2–7)	7 (3–18)	4 (1–11)
Primary diagnosis (ICD-10)						
Substance use disorders (F10–19)	38.7% (1806)	35.7% (859)	33.8% (4,273)	29.7% (1,741)	1.4% (334)	1.3% (212)
Psychotic disorders (F20–29)	2.5% (115)	2.7% (65)	7.4% (935)	8.1% (472)	4.2% (971)	2.9% (456)
Mood disorders (F30–39)	10.7% (502)	11.0% (264)	7.2% (905)	7.4% (435)	28.7% (6671)	28.0% (4436)
Anxiety disorders (F40–48)	13.8% (645)	11.1% (267)	11.2% (1420)	14.6% (852)	15.6% (3618)	16.3% (2,589)
Eating disorders (F50–59)	6.0% (279)	6.0% (145)	1.0% (123)	1.4% (82)	5.6% (1300)	6.6% (1038)
Neurodevelopmental disorders (F80–89)	7.1% (333)	9.4% (225)	5.1% (645)	5.4% (319)	6.8% (1,579)	7.2% (1136)
Behavioural disorders (F90–98)	5.9% (275)	5.7% (137)	2.4% (307)	3.7% (219)	30.1% (6988)	27.4% (4350)
Other F-diagnoses (F00–09, F60–79)	0.8% (39)	0.8% (39)	0.8% (39)	0.8% (39)	1.4% (332)	1.3% (203)
Other non-F/non-Z diagnoses	1.2% (54)	0.6% (15)	1.0% (127)	2.2% (129)	1.9% (440)	2.0% (310)
Z-codes (contact/social reasons)	12.8% (596)	15.9% (382)	22.3% (2821)	18.8% (1098)	3.6% (826)	4.5% (721)
Missing diagnosis	0.6% (28)	0.4% (10)	1.0% (129)	0.1% (8)	0.7% (156)	2.5% (396)

Pre = Jan 1, 2017–June 30, 2020, Post = July 1, 2020–Dec 31, 2021, CAMHS = Child and Adolescent Mental Health Services, IQR = Interquartile range, ICD-10 = 10th revision of the International Classification of Diseases.

Unless otherwise stated, values are percentages with numbers in parentheses indicating number of admissions. Characteristics are reported at the admission level; individuals may contribute more than one admission during the study period

### Primary analyses

**Fig. 1:**
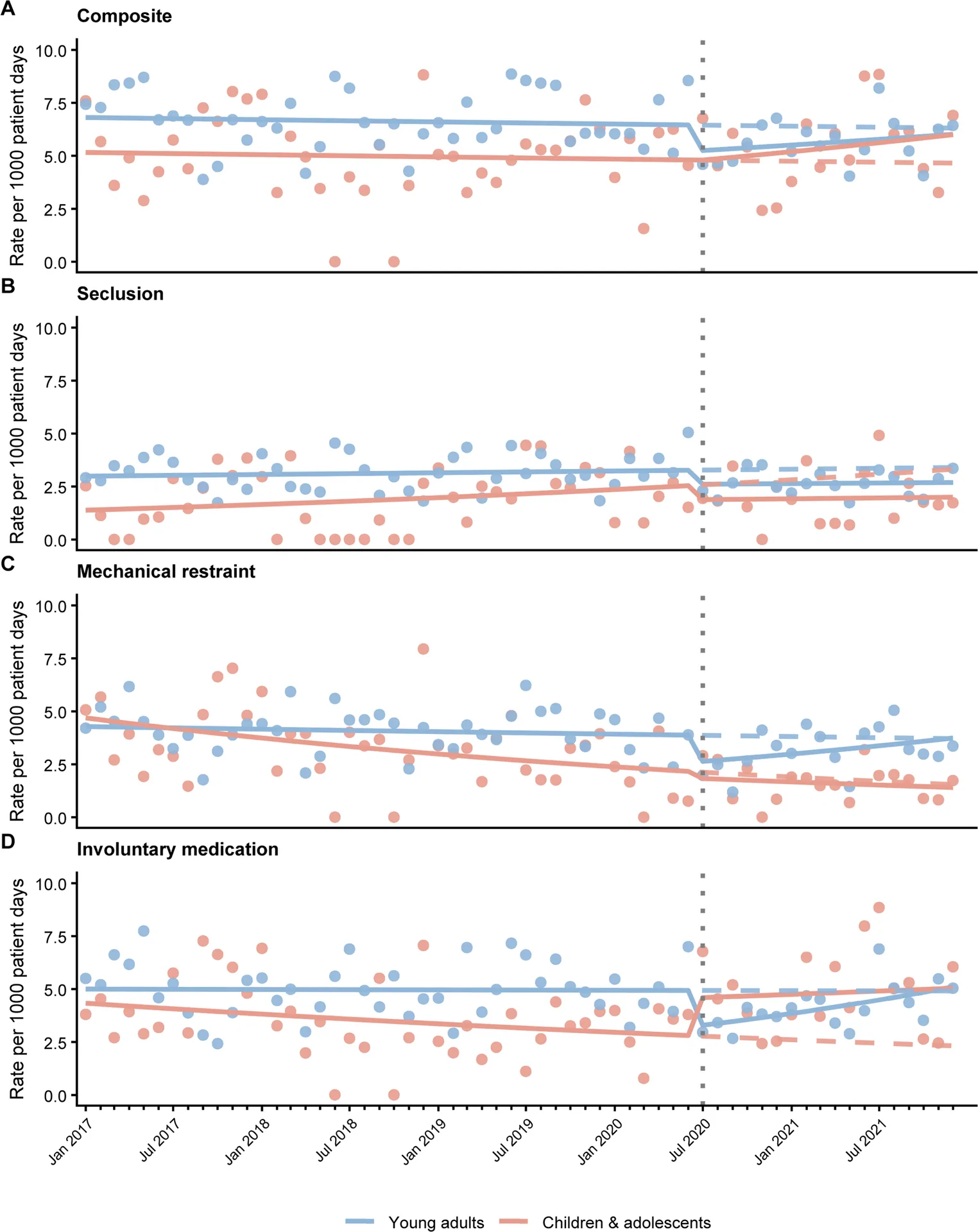
Controlled interrupted time series analysis of psychiatric inpatients subjected to coercive measures. **Notes**: Panels show monthly rates of patients subjected per 1000 patient days for (**A**) a composite outcome (seclusion, mechanical restraint, or involuntary medication), (**B**) seclusion, (**C**) mechanical restraint, and (**D**) involuntary medication Solid lines represent model−estimated trends and dashed lines counterfactual trends. The vertical dotted line indicates the legal change implemented July 1, 2020 Children & adolescents = 0–17−year−olds; young adults = 18–24−year−olds

Figure 1 provides a descriptive overview of time trends, indicating that both baseline levels and pre-intervention slopes differed between minors and young adults across outcomes. Patient-day-standardised simple ITS models suggested no intervention-associated level or slope changes in seclusion, mechanical restraint, or the composite outcome (Table 2). By contrast, involuntary medication among minors appeared to increase immediately post-intervention (incidence rate ratio [IRR] 1.66, 95% CI 1.26–2.19) without evidence of a slope change. Controlled ITS models comparing CAMHS inpatients with young adult psychiatric inpatients in Region Stockholm yielded concordant results, with a larger immediate increase in involuntary medication (IRR 2.49, 95% CI 1.63–3.81) and no significant level or slope changes for other outcomes (Table 2).

**Table 2:** Interrupted time series models of the effect of legal change on psychiatric inpatients subjected to coercive measures in inpatient child and adolescent mental health services: Region Stockholm vs. national Finnish data

	Composite measure			Seclusion			Mechanical restraint			Involuntary medication		
**Patient-day standardised**	**IRR**	**95% CI**	**P-value**	**IRR**	**95% CI**	**P-value**	**IRR**	**95% CI**	**P-value**	**IRR**	**95% CI**	**P-value**
*Simple ITS*												
Level change	1.00	0.81–1.24	0.99	0.73	0.50–1.06	0.099	0.86	0.56–1.33	0.50	1.66	1.26–2.19	**< 0.001**
Slope change	1.02	0.99–1.04	0.16	0.99	0.97–1.01	0.34	1.00	0.98–1.03	0.80	1.02	0.99–1.04	0.22
*Controlled ITS*												
Level change	1.23	0.81–1.88	0.34	0.92	0.50–1.69	0.78	1.26	0.61–2.60	0.53	2.49	1.63–3.81	**< 0.001**
Slope change	1.01	0.97–1.04	0.73	0.99	0.95–1.03	0.58	0.98	0.94–1.03	0.41	0.99	0.96–1.03	0.58
**Population standardised**												
*Simple ITS*												
Level change	0.95	0.77–1.17	0.62	0.69	0.48–0.98	**0.038**	0.81	0.55–1.20	0.29	1.57	1.18–2.09	**0.0021**
Slope change	1.01	0.99–1.03	0.49	0.98	0.96–1.00	0.092	1.00	0.97–1.02	0.81	1.01	0.98–1.03	0.50
*Controlled ITS*												
Level change	1.17	0.77–1.77	0.47	1.01	0.43–2.40	0.97	1.01	0.50–2.04	0.97	··	··	··
Slope change	0.98	0.96–1.01	0.18	0.98	0.92–1.05	0.66	0.96	0.92–1.00	**0.048**	0.94	0.77–1.16	0.58

*ITS* Interrupted time series, *IRR* Incidence Rate Ratio, *CI * Confidence interval

*Composite measure* includes being subjected to seclusion, mechanical restraint or involuntary medication, in Finland (population measures) it also includes physical restraint. All confidence intervals and p-values are based on heteroskedasticity- and autocorrelation-consistent (HAC) standard errors. *Patient-day standardised* uses patient days per month. *Population standardised* uses 0–17-year-olds in population. Controlled ITS compares child and adolescent psychiatric inpatients with young adult psychiatric inpatients (patient-day standardised), and with Finnish CAMHS (population standardised). Bolded figures are significant at *p* < 0.05. The level-change estimate for involuntary medication could not be reliably estimated, likely due to a high proportion of zero-count months in the Finnish data, and is therefore not reported

In population-standardised analyses, neither simple nor controlled ITS models (comparing Region Stockholm CAMHS with Finnish CAMHS), suggested intervention-associated level or slope changes in the any coercive measure composite outcome (Table 2). Simple ITS models indicated an immediate increase in involuntary medication (IRR 1.57, 95% CI 1.18–2.09) and an immediate decrease in seclusion (IRR 0.69, 95% CI 0.48–0.98), with no significant slope change for any outcome. In controlled models, only mechanical restraint showed a reduced post-intervention slope (IRR 0.96, 95% CI 0.92–1.00). Controlled estimates for involuntary medication were not reported due to instability from frequent zero-count months in the Finnish data series. Control-series estimates are provided in Table A2 (Appendix p6).

Exploratory patient-day-standardised event analyses suggested no overall reductions in coercive measure use. In simple ITS models, mechanical restraint decreased immediately, while post-intervention slopes increased for mechanical restraint, involuntary medication, and the composite outcome. In contrast, controlled ITS models indicated an immediate increase in the composite outcome, with no statistically significant intervention-associated level or slope changes for other outcomes (Appendix p8).

### Secondary analyses

Mean duration analyses of coercive measures in Region Stockholm found significantly increased post-intervention slopes among minors relative to young adults for seclusion (mean duration ratio 1.12, 95% CI 1.07–1.18), mechanical restraint (1.06, 95% CI 1.01–1.11), and the combined outcome (1.08, 95% CI 1.04–1.13), consistent with increasing mean durations in minors despite declining durations in young adults (Table 3; Appendix p9 for time series plots).

**Table 3:** Controlled interrupted time series models of effects of legal change on mean duration of coercive measures in Region Stockholm inpatient child and adolescent mental health services

	Combined			Seclusion			Mechanical restraint		
	**Ratio**	**95% CI**	**p-value**	**Ratio**	**95% CI**	**p-value**	**Ratio**	**95% CI**	**p-value**
Level change	0.86	0.41–1.81	0.69	0.90	0.44–1.85	0.77	0.73	0.43–1.25	0.25
Slope change	1.08	1.04–1.13	**< 0.001**	1.12	1.07–1.18	**< 0.001**	1.06	1.01–1.11	**0.012**

*CI *Confidence interval. Combined durations calculated the mean durations of both seclusion and mechanical restraint per month. Estimates for intervention effects are expressed as between-group contrasts relative to the reference group (young adult psychiatric inpatients). All confidence intervals and p-values are based on heteroskedasticity- and autocorrelation-consistent (HAC) standard errors. Mean duration ratios were estimated using log-linear interrupted time series models and exponentiated to obtain mean duration ratios. Bolded figures are significant at *p* < 0.05

Sedative medication doses per patient-days showed no statistically significant intervention-associated level or slope changes overall or by subgroup (Table 4; Appendix pp7, 10–11). When modelled as proportions of patients, no immediate level changes were observed; however, small post-intervention slope decreases were seen for sedatives overall and across most subgroups, with exceptions in specific antipsychotic subgroups (Table 4, Appendix pp7, 10–11).

**Table 4:** Controlled interrupted time series models of effect of legal change on sedative medication use in Region Stockholm inpatient child and adolescent mental health services

	All sedatives			Antipsychotics		
**Doses per patient-days**	**IRR**	**95% CI**	**p-value**	**IRR**	**95% CI**	**p-value**
Level change	0.91	0.79–1.06	0.22	0.85	0.63–1.16	0.31
Slope change	1.00	0.99–1.01	0.71	0.99	0.98–1.01	0.38
Patients with sedative medication	**OR**	**95% CI**	***p*-value**	**OR**	**95% CI**	***p*-value**
Level change	1.13	0.90–1.40	0.29	0.97	0.71–1.33	0.86
Slope change	0.98	0.96–1.00	**0.048**	0.99	0.98–1.01	0.41

*IRR *incidence rate ratio, *OR *odds ratio,* CI *confidence interval

Estimates for intervention effects are expressed as between-group contrasts relative to the reference group (young adult psychiatric inpatients). All confidence intervals and p-values are based on heteroskedasticity- and autocorrelation-consistent (HAC) standard errors. ATC classification of sedative medications: All sedatives = N05A, N05B, N05C, R06AD01 (alimemazine) and R06AD02 (prometazine); Antipsychotics = N05A

### Sensitivity analyses

Sensitivity analyses supported the robustness of findings for CAMHS inpatients’ coercive measure exposure: excluding eating disorder admissions, using admission month to align event timing, and alternative duration models (geometric means, multiple imputation), by yielding results consistent with primary analyses (Appendix pp12–13, 15). Negative exposure control analyses found no spurious level changes; however, it suggested modest background downward trends for most sedative medication outcomes (Appendix p16). Results were also robust to an 18-month Newey-West/HAC lag specification, with conclusions unchanged, apart from evidence of an immediate level decrease in seclusion in the simple ITS model (Appendix p14).

## Discussion

In this controlled interrupted time series study, we found no coherent evidence of reduced coercive measure use in CAMHS inpatient care following Sweden’s 2020 legislative reform. Instead, contrary to reform objectives, results indicated an immediate up to two-fold increase in involuntary medication use, alongside upward trends in the duration of mechanical restraint and seclusion compared to young adults. Evidence of reduction was limited: seclusion decreased only in the uncontrolled population-standardised models, and mechanical restraint showed a small relative post-intervention decline only in the controlled comparison with Finnish CAMHS.

The immediate increase in involuntary medication use may reflect initial clinical uncertainty following the legal change. In our prior study of staff experiences with this legislative reform [9], clinicians described substantial apprehension about managing difficult situations under tighter restrictions, with some reporting increased reliance on involuntary medication as a compensatory strategy to prevent or shorten mechanical restraint and seclusion episodes. Without a clear change in the composite outcome, the rise in involuntary medication is more consistent with increased use alongside other coercive measures, than with a shift towards increased involuntary medication alone. This may reflect a compensatory response to the uncertainty and perceived constraints of the revised legal framework. The immediate post-reform period could thus capture both the reform itself and a transient implementation phase. Excluding it might better estimate a more stable medium-term effect, but would also omit part of the reform’s real-world impact on young people receiving inpatient care during implementation. 

Apparent reductions in seclusion were not consistent across analyses; appearing only in uncontrolled population-standardised models (and under a longer HAC lag) but not in patient-day-standardised models. This likely reflects different estimands: population-standardised rates quantify population burden, whereas patient-day-standardised rates capture coercion per unit inpatient time. Overall, results varied across outcomes and specifications, providing no coherent evidence of a sustained reduction. Exploratory event-based analyses likewise suggested no overall reduction in coercive measure use. Although some level and slope changes were observed, event rates are sensitive to recording practices and did not alter the primary conclusion.

Despite the immediate increase in involuntary medication, we found no reform-associated increases in overall sedative medication use. Negative exposure control analyses suggested consistent downward secular trends across most sedative medication groups, indicating greater concentration of pharmacological management within coercive episodes than on overall sedative medication use.

The post-reform increase in mean duration of seclusion and mechanical restraint is paradoxical, given that the reform introduced shorter mandatory reassessment intervals intended to facilitate earlier termination. Several factors could explain this. First, the “pressure of the clock” may increase environmental stress and impede staff de-escalation, prolonging crisis states. Second, if involuntary medication is increasingly used alongside other coercive measures as a compensatory strategy, this might hinder de-escalation and prolong coercive episodes. Third, once the legal thresholds for coercive measures are met, renewals may become routine administrative procedures rather than genuine prompts for termination, as suggested by qualitative findings on perceived administrative burden with limited clinical relevance [9].

The immediate level reductions in the young adult control series are plausibly related to system-wide disruptions around the onset of the COVID-19 pandemic. Swedish national data indicate that fewer adults sought psychiatric emergency care during the pandemic, while a higher proportion of those who did present were admitted, including under compulsory care; suggesting more severe presentations among attendees rather than increased severe mental illness in the general population [15]. This aligns with Danish register evidence of reduced acute psychiatric contacts following the March 2020 lockdown [16], and inpatient studies reporting increased symptom severity at admission during the pandemic in both adolescents and adults [17, 18]. While greater clinical severity might be expected to increase coercion, immediate level changes in the control series were consistently downward, suggesting concurrent changes in service delivery or practice may have influenced coercive use independently of patient acuity. In contrast, the immediate increase in involuntary medication among minors was consistent across specifications and not mirrored in Finland, making a purely pandemic-related explanation less likely.

Our finding of no clear reduction in coercive measure use aligns with some, but not all, prior legislative reform evaluations. Reports from France and Germany similarly found no reform-associated changes in a single centre [6], or in residential institutions for youth with intellectual disabilities [7]. However, most previous studies lacked control groups and were therefore less able to distinguish reform effects from concurrent exposures or secular trends.

The observed increases in involuntary medication and longer durations are compatible with evidence that legal constraints may shift the type and intensity of coercive practices rather than reduce overall exposure. Following legislative reforms in Dutch and German adult psychiatry [3, 4], studies reported decreased use of some coercive measures alongside increased use of others, suggesting substitution rather than reduction. The strongest causal evidence comes from a German natural experiment, where seclusion and restraint increased 28% during a temporary medication ban [2], suggesting that restricting one measure might drive compensatory increases in alternatives.

In contrast, Ireland reported substantial declines in restrictive practices following revised Rules and Code of Practice in 2023 [8]. Reductions were particularly pronounced in CAMHS centres, where physical restraint and seclusion episodes fell 53% and 80% respectively, alongside decreased duration of both measures.

Importantly, Ireland’s reform combined revised rules with real-time reporting, enhanced regulatory oversight, post-incident reviews, and staff training; a multicomponent intervention rather than legislative change alone. Still, without a control group, observed declines may reflect secular trends rather than reform impact. A recent systematic review found the strongest evidence for reducing coercive measures comes from such multicomponent interventions targeting organisational culture and staff practices [19]. Taken together, legislation alone, without organisational development, staff training, and implementation support, may risk unintended substitution effects or, as in the present study, maintained coercion levels.

### Strengths and limitations

This study’s primary strengths include the controlled interrupted time series design with dual comparators (within-region young adults and cross-national Finnish data), granular electronic medical records, incorporation of lived experience perspectives, and robust findings across extensive sensitivity analyses.

Several limitations should be noted. The legal reform coincided with the COVID-19 pandemic; although two control groups mitigate confounding, differential pandemic effects across age groups and countries cannot be excluded. The concurrent incorporation of the UN Convention on the Rights of the Child into Swedish law may have influenced practice independently of the reform, and its effects cannot be isolated in this design. Post-intervention follow-up was limited to 18 months, and longer observation might be needed to assess sustainability after services adapt to the reform. Although not observed or reported in our qualitative study [9], documentation practices may have changed around implementation, potentially introducing differential measurement error in exposure to or duration of coercive measures. Reliance on Region Stockholm data rather than national Swedish data may limit generalisability, although Stockholm comprises 24% of Sweden’s paediatric population. Several limitations affected the comparison with Finland: data structure differed from Swedish records (coercive measures recorded per admission without exact timestamps), and data access restrictions for individual coercive measures required separate rather than unified modelling. This assumed independence when combining standard errors, potentially yielding conservative inference if trends were correlated across countries. Finally, data quality concerns regarding documentation of individual coercive measure use, forced us to deviate from preregistration by using only the more reliable and detailed Region Stockholm data.

In conclusion, the 2020 Swedish legislative reform did not reduce coercive measure use in inpatient CAMHS; instead, it was associated with increased involuntary medication and longer durations of restraint and seclusion. These findings underscore that legislation alone might be insufficient to reduce coercive practices without parallel organisational and cultural change. 

### Lived experience commentary by Josefin Andersson, Liv Fischer and OD (anonymity preferred), young adults from Sweden with previous experience of compulsory care and coercive measures in inpatient child and adolescent mental health services.

This study evaluates a legal reform intended to reduce coercive measures in inpatient child and adolescent mental health services, yet the findings do not suggest a clear reduction. From our lived experience, this is not surprising. On the ward, the effect of formal rules and legislation can feel secondary to staffing levels, time pressure, and the local culture among staff. Coercive practices may differ markedly between units and even between shifts, depending on who is present and how the law is interpreted at the moment. When services are under strain, staff can fall back on familiar routines, and newer staff trying to work differently may struggle to alter established approaches. We also note that the study points towards longer mean durations after the reform, rather than the expected shortening. This echoes our lived experience that coercive episodes can become prolonged when distress escalates and staff have limited time, capacity, and access to workable alternatives.

Above all, what we experienced as decisive was whether staff could create a trustworthy environment where you feel believed, cared for, and confident that interventions are truly in your best interests. That kind of culture cannot be legislated into existence, and it does not follow automatically from revised routines. Youth should also be informed about legal changes, so they understand time limits, decisions, and their rights, and can better retain dignity and a sense of control. For us, the key issue is not only removing coercion, but replacing it with credible alternatives, such as staff training, rehearsed de-escalation strategies, and treatment that builds trust.

## Supplementary Information

Below is the link to the electronic supplementary material.

Supplementary Material 1 (PDF 964 KB) 

## Data Availability

Due to national regulations and data protection legislation, the authors are not permitted to distribute data to third parties. Researchers wishing to replicate the analyses presented in this study may submit an application for individual-level data access directly to the respective data holders.
