# Supplementary material for: Impact of legislative reform on coercive measure use in child and adolescent psychiatry: A controlled interrupted time series analysis
Source: Eur Child Adolesc Psychiatry. 2026 May 18;35(9):2853–63. doi: 10.1007/s00787-026-03035-1 (PMC13695617; doi:10.1007/s00787-026-03035-1)
Supplement: Supplementary file 1 [file 787_2026_3035_MOESM1_ESM.pdf]

Supplementary materials

Supplementary materials to "Impact of legislative reform on coercive measure use in child and adolescent psychiatry: A controlled time series analysis" by Astrid Moell, Erik Pettersson, Riittakerttu Kaltiala & Niklas Långström in *European Child & Adolescent Psychiatry*.

Corresponding author: Astrid Moell, Centre for Psychiatry Research, Department of Clinical Neuroscience, Karolinska Institutet, & Stockholm Health Care Services, Region Stockholm, Sweden. astrid.moell@ki.se

Content

Additional details on settings and methods 2

Table S1. Key child-specific amendments to the Swedish Compulsory Psychiatric Care Act (effective July 1, 2020). 2

Table S2. Control-series estimates of ITS analyses in young adult psychiatric inpatients in Region Stockholm and Finnish CAMHS. 6

Table S3. Controlled ITS models of the effect of legal change of sedative medication use in Region Stockholm inpatient CAMHS. 7

Table S4. Exploratory ITS analyses of the effect of legal change on event-related patient day standardised coercive measure rates in Region Stockholm inpatient CAMHS. 8

Supplementary figures (secondary analyses) 9

Table S5. Sensitivity analyses of controlled ITS models of effect of legal change on patients subjected to coercive measures per patient days in Region Stockholm inpatient CAMHS. 12

Table S6. Sensitivity analyses using admission month as event month in controlled ITS models of effect of legal change on patients subjected to coercive measures per population in Region Stockholm inpatient CAMHS. 13

Table S7. Sensitivity analysis using Newey-West HAC standard errors with alternative lag structures (12-month vs 18-month lag) for patient-day standardised models 14

Table S8. Sensitivity analyses of controlled ITS models of the effect of legal change on duration ratios of mechanical restraint and seclusion in Region Stockholm inpatient CAMHS. 15

Table S9. Sensitivity analyses of controlled ITS models of the effect of legal change on sedative medication use in Region Stockholm inpatient CAMHS. 16

Additional details on settings and methods

Judicial frameworks in Sweden and Finland

In Sweden, involuntary psychiatric care is regulated by the Compulsory Psychiatric Care Act (Lagen om psykiatrisk tvångsvård, 1991:1128).[1] Compulsory admission requires that the patient (a) suffers from a severe mental disorder, (b) has an indispensable need for inpatient psychiatric care, and (c) opposes the proposed care or there is reason to assume that care cannot be provided voluntarily. These criteria apply equally to adults (18+ years) and to children and adolescents (0-17 years).

Coercive measures may only be used for patients who are involuntarily hospitalised. Legally regulated coercive measures include seclusion, mechanical restraint, and treatment without consent. Treatment without consent is not further specified in the Act; however, in clinical practice it primarily refers to involuntary medication, typically administered as intramuscular injections under physical holding or brief mechanical restraint. Forced nasogastric feeding and, in exceptional cases, electroconvulsive therapy may also be administered under the category of treatment without consent. Further, inspecting shipments and property, taking possession of property, body search, and limitations in electronic communications are also coercive measures regulated by law. Physical holding is permitted only as a short-term measure to enable other protective or coercive interventions and is not registered as a separate coercive measure.

In *Finland,* involuntary psychiatric care is regulated by the Mental Health Act (Mielenterveyslaki, 1116/1990).[2] Involuntary admission requires that the patient suffers from (a) mental illness (=psychosis) (adults 18+) or a severe mental disorder (minors), so that (b) without immediate treatment their mental illness / severe mental disorder would deteriorate or endanger their health or safety or health and safety of others, and (c) other treatment options are not suitable for use. If involuntarily hospitalised, minors have to be treated separate from adults.

Legally regulated coercive measures include seclusion, mechanical restraint, physical restraint, and treatment without consent, including involuntary medication, inspecting shipments and property, taking possession of property, body search and restricted leave . These measures may only be applied to involuntarily hospitalised patients and must always be proportionate, necessary, and time-limited. The coercive measures have the same definitions as in Sweden's Compulsory Psychiatric Care Act apart from *physical restraint* which is a coercive measure in itself. Physical restraint involves staff physically holding the patient in order to prevent immediate harm or to enable another protective or coercive intervention.

From July 1, 2020, the Swedish Compulsory Psychiatric Care Act was amended for patients under 18 years of age, while remaining unchanged for adults. A summary of the specific legislative changes is provided in Table A1 (adapted from Moell and colleagues (2024)).[3]

Table S1. Key child-specific amendments to the Swedish Compulsory Psychiatric Care Act (effective July 1, 2020).

	**Before July 1, 2020**	**From July 1, 2020 (patients <18 years)**
**Maximum duration per formal decision**	*Seclusion:* up to 8 hours per decision	*Seclusion:* up to 2 hours per decision
	Mechanical restraint: up to 4 hours per decision	*Mechanical restraint:* up to 1 hour per decision
	Limitations in electronic communication: up to 2 months	*Limitations in electronic communication*: up to 1 week
**Legal requisites**	*Seclusion:* Disturbing or aggressive behaviour that seriously disrupts the treatment of other patients	*Seclusion:* Aggressive behaviour that seriously disrupts the treatment of other patients, and only when it is evident that less intrusive measures are insufficient
	Mechanical restraint: Immediate risk of harm to self or others	*Mechanical restraint:* Immediate risk of harm to self, and only when it is evident that less intrusive measures are insufficient
**Reporting to the Health and Social Care Inspectorate**	Prolongation of mechanical restraint or seclusion	Prolongation of mechanical restraint or seclusion, or if three or more separate coercive measure episodes occur during the same admission
**Patient rights and ward conditions**	Information about compulsory care and the right to appeal. Right to an independent support person. Post-measure interview following coercive measures	All previous rights retained, in addition: right to daily activities on the ward, and opportunity to spend at least one hour outdoors per day

Outcomes

Coercive measures were defined in the Region Stockholm dataset in accordance with the Swedish Compulsory Psychiatric Care Act as:

Seclusion: *§20 Seclusion* for both minors and adults pre-legal change, *§20a Seclusion for minors* following legal change. Seclusion entails preventing the patient from moving freely in the ward, usually by locking them in a room, but can also be effected through separating a section of the ward to the patient alone.

Mechanical restraint: *§19 Mechanical restraint* for both minors and adults pre-legal change, *§19a Mechanical restraint for minors* following the legal change. Mechanical restraint entails using a belt bed with five or seven cuffs/belts to restrain the individual to the bed and prevent them from moving freely.

Involuntary medication: *§17 Treatment against the patient's will*, in the Region Stockholm dataset, was recorded in different categories, of which one was *§17 Medication against one's will*. Involuntary medication entails medication used against the patient's will, usually by staff physically holding the patient down, and an intramuscular injection is given. The reason for using involuntary medication is not specified in the registration; using it for rapid control of a patient in distress, or for non-acute involuntary treatment with long-acting injections, are registered the same way.

In the Finnish dataset, coercive measures were defined in accordance with the Finnish Mental Health Act as:

Seclusion: was defined as secluding a patient from other patients against their will under *§22*. Seclusion may be used if the patient (1) would probably harm themself or others based on behaviour or threats, (2) seriously hampers others' care or seriously jeopardises their own safety or would probably cause significant property damage, or (3) isolation is necessary for other especially substantial therapeutic reasons.

Mechanical restraint: Mechanical restraint was defined as tying down a patient using belts or a comparable method under *§22*, permitted if other measures are not sufficient.

Physical restraint: Physical restraint was defined as forcible holding of the patient. Under *§22* staff may use necessary coercive means to hold the patient in order to seclude them (with immediate notification to the attending physician), and *§22* further permits holding in other situations if necessary for therapeutic reasons. In addition, *§22* states that the attending physician decides on examinations/treatments given regardless of the patient's will and may decide on "holding or tying down" and comparable short-term measures necessary to deliver treatment.

Involuntary medication: *§22* provides that the attending physician decides on treatments and examinations given regardless of the patient's will and on necessary short-term limitation measures (including holding/tying down) to deliver treatment.

We deviated from the pre-registered protocol by omitting nasogastric forced feeding from the primary composite outcome. Given the smaller sample size of the Region Stockholm cohort compared to the anticipated national Swedish dataset, not possible to employ here, preliminary data inspection suggested that forced feeding was limited to a few recurring individuals. Retaining nasogastric forced feeding would have introduced unnecessary noise and potentially biased the monthly patient-based rates.

Children and adolescent psychiatric inpatients

Forensic psychiatric services were excluded from the Stockholm analyses but retained in the Finnish data because of clinical differences. In Region Stockholm, inclusion of forensic services would have disproportionately weighted the dataset with young adult forensic patients characterised by very long periods of care and repeated use of coercive measures, while very few minors (<five individuals per year nationwide) receive forensic psychiatric care. In Sweden, forensic psychiatric services are restricted to individuals sentenced to compulsory forensic care following serious criminal offences committed under the influence of severe mental illness, or to individuals temporarily requiring psychiatric care for a severe mental illness while detained on the order of a public prosecutor. In Finland, by contrast, adolescent forensic psychiatric care includes both court-ordered compulsory

treatment and long-term specialised care for adolescents whose needs cannot be met in regular child and adolescent psychiatric units, typically due to early-onset psychotic disorders or severe autism with violent behaviour.

Missing data: Region Stockholm

When calculating patient days per month, missing admission (n = 102) or discharge (n = 198) dates were supplemented using inpatient medication timestamps, reducing missingness to 18 admissions and 9 discharges. For admissions without medication administration data (n = 25), admission periods were imputed as up to 30 days, truncated at study boundaries or at subsequent admission dates where applicable. This window was chosen because missingness was concentrated at the study's start (early 2017) and end (late 2021), suggesting these were long-term admissions truncated by the employed observation period. Remaining cases were handled via complete-case analysis.

For coercive measure data, 3.7% of events had a missing start time (200/5450) and were excluded as temporal placement could not be reliably determined. Stop time was missing for 5.8% of events (315/5450), these events were included in the final analyses of counts (patients subjected per month) as these represented documented measures with known start dates. Logistic regression indicated missingness in stop time decreased over time and was higher for younger patients and seclusion events. Primary analyses of durations used complete-case data.

Details on sensitivity analyses: Region Stockholm data

Two sensitivity analyses were performed to assess robustness for coercive measure duration models: using an alternative estimator, and multiple imputation. The first sensitivity analysis reanalysed complete cases using monthly geometric mean durations (reducing the influence of extreme values) instead of arithmetic means. Both approaches used log-transformed monthly aggregates in interrupted time series models, with results back-transformed for easier interpretation.

For the second analyses, missing duration data were imputed directly using multiple imputation by chained equations using R package 'mice',[4] with predictive mean matching (m=40). To account for the right-skewed distribution and improve model performance, imputation was conducted on the log scale, and included event start time-derived calendar variables, time since admission, age, sex, age group, diagnosis, event category (mechanical restraint vs seclusion), and patient/admission identifiers as auxiliary variables. For both geometric mean and multiple imputation analyses, estimates were back-transformed to the original scale for interpretability.

Sedative medications: Region Stockholm data

We divided sedative medications into clinically meaningful groups:

**Sedative medication group**	**ATC code**
All sedatives	N05A, N05B, N05C, R06AD01 (alimemazine) and R06AD02 (prometazine)
Antipsychotics	N05A
All sedatives except melatonin	N05A, N05B, N05C, R06AD01 and R06AD02, excluding N05CH01(melatonin)
All sedatives except antipsychotics	N05B, N05C, R06AD01 and R06AD02N05
All sedative except antipsychotics and melatonin	N05B, N05C, R06AD01 and R06AD02N05, excluding N05CH01
Antipsychotics except lithium	N05A excluding N05AN01 (lithium)

References

1 Lagen om psykiatrisk tvångsvård [Compulsory Psychiatric Care Act]. Sveriges riksdag (Svensk författningssamling, SFS), 1991 https://www.riksdagen.se/sv/dokument-lagar/dokument/svensk-forfattningssamling/lag-19911128-om-psykiatrisk-tvangsvard_sfs-1991-1128.

2 Mielenterveyslaki [Mental Health Act]. Finlex (Ministry of Justice, Finland), 1990 https://www.finlex.fi/fi/lainsaadanto/1990/1116.

3 Moell A, Rozental A, Buchmayer S, Kaltiala R, Långström N. Effects of stricter legislation on coercive measures in child and adolescent psychiatric care: A qualitative interview study with staff. *BMC Psychiatry* 2024; **24**: 102.

4 Buuren SV, Groothuis-Oudshoorn K. mice: Multivariate Imputation by Chained Equations in R. *J Stat Softw* 2011; **45**. DOI:10.18637/jss.v045.i03.

Table S2. Control-series estimates of ITS analyses in young adult psychiatric inpatients in Region Stockholm and Finnish CAMHS.

	Composite measure			*Seclusion*			*Mechanical restraint*			*Involuntary medication*		
	**IRR**	**95% CI**	**p-value**	**IRR**	**95% CI**	**p-value**	**IRR**	**95% CI**	**p-value**	**IRR**	**95% CI**	**p-value**
**Young adults**												
Level change	0.81	0.67–0.98	**0.033**	0.80	0.63–1.00	0.053	0.68	0.51–0.92	**0.011**	0.67	0.55–0.81	**<0.0001**
Slope change	1.01	1.00–1.02	0.13	1.00	0.99–1.01	0.96	1.02	1.00–1.05	0.087	1.03	1.02–1.04	**<0.0001**
**Finnish CAMHS**												
Level change	0.81	0.57–1.16	0.26	0.68	0.31–1.48	0.33	0.80	0.45–1.42	0.44	··	··	··
Slope change	1.03	1.01–1.04	**0.0041**	1.00	0.93–1.06	0.92	1.04	1.01–1.07	**0.020**	1.07	0.87–1.31	0.52

Footnotes:
ITS = interrupted time series, CAMHS = child and adolescent mental health services, IRR = incidence rate ratio, CI = confidence interval.
Offset for the young adult models were patient days per month, Finnish CAMHS models used 0–17-year-olds in population. The composite measure includes seclusion, mechanical restraint and involuntary medication, and in Finland physical restraint. All confidence intervals and p-values are based on heteroskedasticity- and autocorrelation-consistent (HAC) standard errors. Bolded figures are significant at $p<0.05$. The level-change estimate for involuntary medication in Finnish CAMHS could not be reliably estimated, likely due to a high proportion of zero-count months and is therefore not reported.

Table S3. Controlled ITS models of the effect of legal change of sedative medication use in Region Stockholm inpatient CAMHS.

	All sedatives			*Antipsychotics*			*All sedatives excl. melatonin*			*All sedatives excl. antipsychotics*			*All sedatives excl. antipsychotics & melatonin*			*Antipsychotics excl. lithium*		
**Doses per patient-days**																		
	**IRR**	**95% CI**	**p-value**	**IRR**	**95% CI**	**p-value**	**IRR**	**95% CI**	**p-value**	**IRR**	**95% CI**	**p-value**	**IRR**	**95% CI**	**p-value**	**IRR**	**95% CI**	**p-value**
Level change	0.91	0.79–1.06	0.22	0.85	0.63–1.16	0.31	0.86	0.73–1.02	0.074	0.96	0.77–1.19	0.69	0.87	0.70–1.08	0.20	0.89	0.64–1.23	0.47
Trend change	1.00	0.99–1.01	0.71	0.99	0.98–1.01	0.38	1.00	0.99–1.01	0.55	1.01	0.99–1.03	0.42	1.01	0.99–1.03	0.24	0.99	0.97–1.01	0.14
**Proportion of patients**																		
	**OR**	**95% CI**	**p-value**	**OR**	**95% CI**	**p-value**	**OR**	**95% CI**	**p-value**	**OR**	**95% CI**	**p-value**	**OR**	**95% CI**	**p-value**	**OR**	**95% CI**	**p-value**
Level change	1.13	0.90–1.40	0.29	0.97	0.71–1.33	0.86	1.02	0.79–1.32	0.88	1.08	0.94–1.26	0.24	0.99	0.83–1.21	0.99	0.97	0.71–1.34	0.90
Slope change	0.98	0.96–1.00	**0.048**	0.99	0.98–1.01	0.41	0.98	0.96–1.00	**0.028**	0.98	0.96–0.99	**0.00058**	0.97	0.96–0.98	**<0.0001**	0.99	0.98–1.01	0.28

Footnotes:

ITS = interrupted time series, IRR = incidence rate ratio, CAMHS = child and adolescent mental health services, OR = odds ratio, CI = confidence interval.

Estimates for intervention effects are expressed as between-group contrasts relative to the reference group (young adult psychiatric inpatients). All confidence intervals and p-values are based on heteroskedasticity- and autocorrelation-consistent (HAC) standard errors. Bolded figures are significant at $p<0.05$. ATC classification of sedative medications: All sedatives = N05A, N05B, N05C, R06AD01 (alimemazine) and R06AD02 (prometazine); Antipsychotics = N05A; All sedatives except melatonin = N05A, N05B, N05C, R06AD01 and R06AD02, excluding N05CH01 (melatonin); All sedatives except antipsychotics = N05B, N05C, R06AD01 and R06AD02N05; All sedatives except antipsychotics and melatonin = N05B, N05C, R06AD01 and R06AD02N05, excluding N05CH01; Antipsychotics except lithium = N05A excluding N05AN01 (lithium).

Table S4. Exploratory ITS analyses of the effect of legal change on event-related patient day standardised coercive measure rates in Region Stockholm inpatient CAMHS.

	Composite measure			*Seclusion*			*Mechanical restraint*			*Involuntary medication*		
	**IRR**	**95% CI**	**p-value**	**IRR**	**95% CI**	**p-value**	**IRR**	**95% CI**	**p-value**	**IRR**	**95% CI**	**p-value**
**Simple ITS**												
Level change	1.19	0.83–1.72	0.35	2.05	0.98–4.29	0.057	0.44	0.27–0.71	**0.00079**	1.19	0.70–2.02	0.52
Slope change	1.03	1.01–1.06	**0.0051**	0.96	0.91–1.02	0.21	1.07	1.02–1.12	**0.0027**	1.05	1.01–1.08	**0.012**
**Controlled ITS**												
Level change	1.98	1.28–3.09	**0.0023**	2.67	0.98–7.29	0.056	0.92	0.49–1.71	0.78	1.85	0.99–3.46	0.054
Slope change	1.01	0.98–1.04	0.61	0.96	0.87–1.06	0.40	1.03	0.99–1.07	0.12	1.03	0.98–1.08	0.31

Footnotes:
ITS = interrupted time series, CAMHS = child and adolescent mental health services, IRR = incidence rate ratio, CI = confidence interval.
The composite measure includes seclusion, mechanical restraint and involuntary medication. Estimates for intervention effects are expressed as between-group contrasts relative to the reference group (young adult psychiatric inpatients). All confidence intervals and p-values are based on heteroskedasticity- and autocorrelation-consistent (HAC) standard errors. Bolded figures are significant at $p<0.05$.

Supplementary figures (secondary analyses)

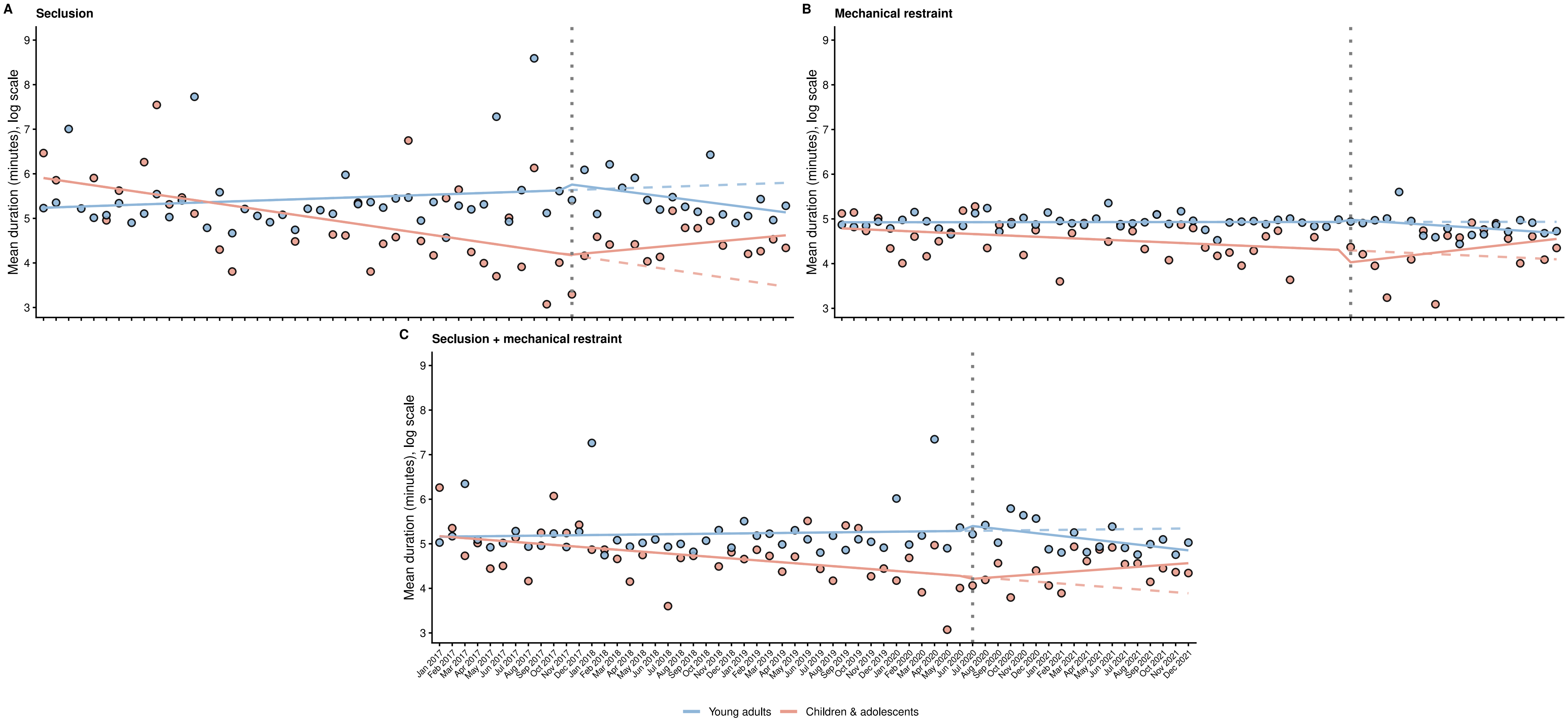

Figure S1. Controlled interrupted time series analysis of log mean duration of coercive measures.

Notes: Panels show log mean duration (minutes) per month. Combined duration was calculated as the mean duration (minutes) across all seclusion and mechanical restraint events within each month. Solid lines represent model−estimated trends and dashed lines counterfactual trends.

The vertical dotted line indicates the legal change implemented July 1, 2020. Children & adolescents = 0–17−year−olds; young adults = 18–24−year−olds.

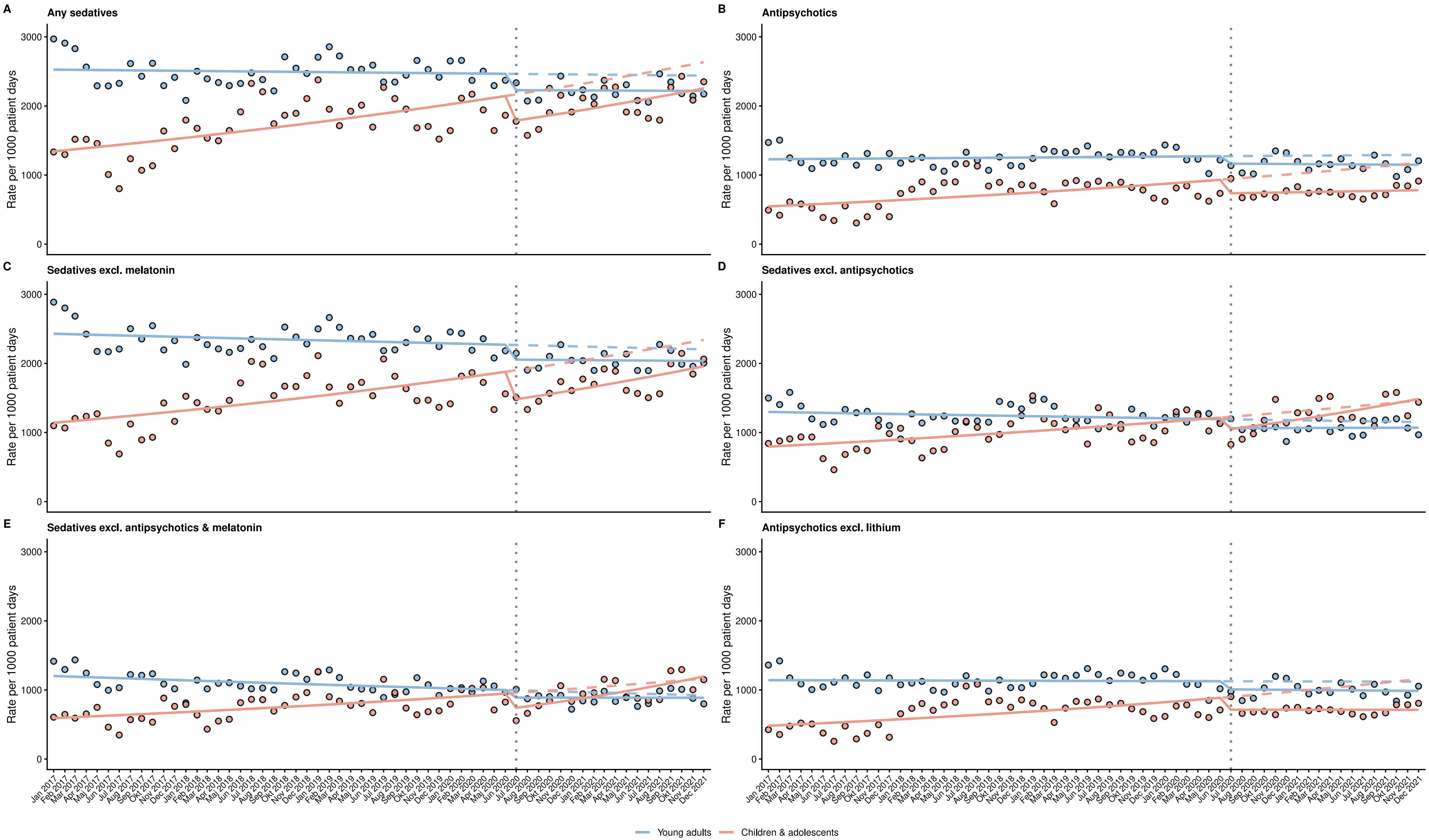

Figure S2. Controlled interrupted time series analysis of sedative medication dosing rates.

Notes: Panels show monthly proportions for six medication groups. Panel A: ATC N05 (antipsychotics, anxiolytics, hypnotics/sedatives), R06AD01 (Alimemazine) and R06AD02 (Prometazine); panel B: N05A (Antipsychotics), panel C: N05 excl. N05CH01 (Melatonin); Panel D: N05 excl. N05A; Panel E: N05 excl. N05A & N05CH01; Panel F: N05 excl. N05AN01 (Lithium).

Solid lines represent model−estimated trends and dashed lines counterfactual trends. The vertical dotted line indicates the legal change implemented July 1, 2020. Children & adolescents = 0–17−year−olds; young adults = 18–24−year−olds.

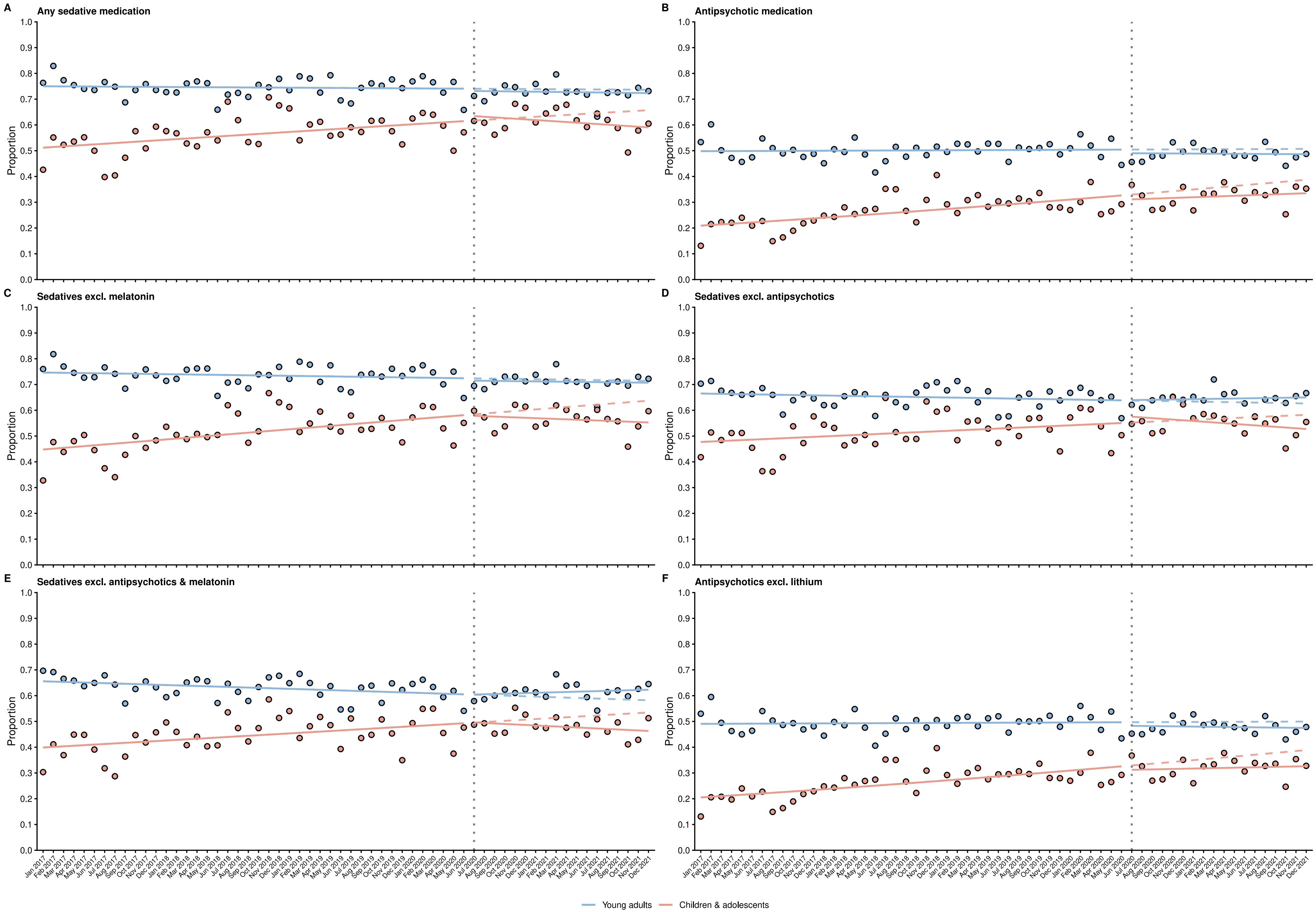

Figure S3. Controlled interrupted time series analysis of sedative medication use among psychiatric inpatients.

Notes: Panels show monthly proportions for six medication groups. Panel A: ATC N05 (antipsychotics, anxiolytics, hypnotics/sedatives), R06AD01 (Alimemazine) and R06AD02 (Prometazine); panel B: N05A (Antipsychotics), panel C: N05 excl. N05CH01 (Melatonin); Panel D: N05 excl. N05A; Panel E: N05 excl. N05A & N05CH01; Panel F: N05 excl. N05AN01 (Lithium).

Solid lines represent model−estimated trends and dashed lines counterfactual trends. The vertical dotted line indicates the legal change implemented July 1, 2020. Children & adolescents = 0–17−year−olds; young adults = 18–24−year−olds.

Table S5. Sensitivity analyses of controlled ITS models of effect of legal change on patients subjected to coercive measures per patient days in Region Stockholm inpatient CAMHS.

	Composite measure			*Seclusion*			*Mechanical restraint*			*Involuntary medication*		
	**IRR**	**95% CI**	**p-value**	**IRR**	**95% CI**	**p-value**	**IRR**	**95% CI**	**p-value**	**IRR**	**95% CI**	**p-value**
**A. Negative exposure control**												
Level change	0.76	0.38–1.54	0.45	0.78	0.30–2.05	0.61	0.96	0.48–1.93	0.91	0.53	0.22–1.27	0.16
Slope change	1.01	0.95–1.07	0.81	0.99	0.89–1.09	0.78	0.98	0.93–1.04	0.59	1.01	0.95–1.08	0.72
**B. Excluding eating disorders**												
Level change	1.20	0.75–1.91	0.45	0.81	0.46–1.44	0.48	1.25	0.66–2.36	0.50	2.37	1.42–3.97	**0.0010**
Slope change	1.02	0.99–1.06	0.21	0.99	0.95–1.04	0.77	1.00	0.97–1.04	0.91	1.00	0.96–1.05	0.86

Footnotes:
ITS = interrupted time series, IRR = incidence rate ratio, CAMHS = child and adolescent mental health services, CI = confidence interval.
The composite measure includes being subjected to seclusion, mechanical restraint or involuntary medication. Estimates for intervention effects are expressed as between-group contrasts relative to the reference group (young adult psychiatric inpatients). All confidence intervals and p-values are based on heteroskedasticity- and autocorrelation-consistent (HAC) standard errors. The negative exposure control (A) used a placebo intervention date of July 1, 2018. The exclusion analysis (B) removed all inpatient admissions with a main diagnosis of an eating disorder (ICD-10 F50). Bolded figures are significant at $p<0.05$.

Table S6. Sensitivity analyses using admission month as event month in controlled ITS models of effect of legal change on patients subjected to coercive measures per population in Region Stockholm inpatient CAMHS.

	Composite measure			*Seclusion*			*Mechanical restraint*			*Involuntary medication*		
	**IRR**	**95% CI**	**p-value**	**IRR**	**95% CI**	**p-value**	**IRR**	**95% CI**	**p-value**	**IRR**	**95% CI**	**p-value**
Level change	0.92	0.60–1.42	0.72	1.08	0.47–2.49	0.86	0.89	0.45–1.74	0.73	..	..	..
Slope change	0.98	0.95–1.00	0.066	0.98	0.91–1.05	0.55	0.95	0.90–1.00	**0.039**	0.94	0.76–1.15	0.53

Footnotes:
ITS = interrupted time series, IRR = incidence rate ratio, CAMHS = child and adolescent mental health services, CI = confidence interval.
The composite measure includes being subjected to seclusion, mechanical restraint or involuntary medication, in Finland also physical restraint. Estimates for intervention effects are expressed as between-group contrasts relative to the reference group (Finnish CAMHS). All confidence intervals and p-values are based on heteroskedasticity- and autocorrelation-consistent (HAC) standard errors. Bolded figures are significant at $p<0.05$. The level-change estimate for involuntary medication could not be reliably estimated, likely due to the high proportion of zero-count months in the Finnish data, and is therefore not reported

Table S7. Sensitivity analysis using Newey-West HAC standard errors with alternative lag structures (12-month vs 18-month lag) for patient-day standardised models

				Lag 12		Lag 18	
**Model**	**Outcome**	**Parameter**	**IRR**	**95% CI**	**p-value**	**95% CI**	**p-value**
Simple ITS	Composite measure	Level change	1.00	0.81–1.24	0.99	0.82–1.22	0.99
Simple ITS	Composite measure	Slope change	1.02	0.99–1.04	0.16	1.00–1.03	0.069
Controlled ITS	Composite measure	Level change	1.23	0.81–1.88	0.34	0.88–1.72	0.23
Controlled ITS	Composite measure	Slope change	1.01	0.97–1.04	0.73	0.98–1.04	0.71
Simple ITS	Seclusion	Level change	0.73	0.50–1.06	0.099	0.56–0.96	**0.023**
Simple ITS	Seclusion	Slope change	0.99	0.97–1.01	0.34	0.97–1.01	0.28
Controlled ITS	Seclusion	Level change	0.92	0.50–1.69	0.78	0.53–1.58	0.75
Controlled ITS	Seclusion	Slope change	0.99	0.95–1.03	0.58	0.96–1.02	0.54
Simple ITS	Mechanical restraint	Level change	0.86	0.56–1.33	0.50	0.61–1.21	0.38
Simple ITS	Mechanical restraint	Slope change	1.00	0.98–1.03	0.80	0.98–1.03	0.76
Controlled ITS	Mechanical restraint	Level change	1.26	0.61–2.60	0.53	0.67–2.36	0.47
Controlled ITS	Mechanical restraint	Slope change	0.98	0.94–1.03	0.41	0.94–1.02	0.37
Simple ITS	Involuntary medication	Level change	1.66	1.26–2.19	**0.00032**	1.27–2.17	**0.00023**
Simple ITS	Involuntary medication	Slope change	1.02	0.99–1.04	0.22	1.00–1.03	0.087
Controlled ITS	Involuntary medication	Level change	2.49	1.63–3.81	**<0.0001**	1.74–3.57	**<0.0001**
Controlled ITS	Involuntary medication	Slope change	0.99	0.96–1.03	0.58	0.96–1.02	0.55

Footnotes: HAC = heteroskedasticity- and autocorrelation-consistent, ITS = Interrupted time series, IRR = Incidence Rate Ratio, CI = Confidence interval.
Composite measure includes being subjected to seclusion, mechanical restraint or involuntary medication. Patient-day standardised uses patient days per month. Controlled ITS compares child and adolescent psychiatric inpatients with young adult psychiatric inpatients (patient-day standardised). Bolded figures are significant at $p<0.05$.

Table S8. Sensitivity analyses of controlled ITS models of the effect of legal change on duration ratios of mechanical restraint and seclusion in Region Stockholm inpatient CAMHS.

	Combined			*Seclusion*			*Mechanical restraint*		
**A. Geometric mean, pooled estimates across multiple imputed datasets**	**Duration ratio**	**95% CI**	**p-value**	**Duration ratio**	**95% CI**	**p-value**	**Duration ratio**	**95% CI**	**p-value**
Level change	0.92	0.62–1.38	0.70	1.05	0.64–1.73	0.85	0.87	0.48–1.58	0.82
Slope change	1.06	1.03–1.10	**0.00094**	1.10	1.05–1.16	**0.00011**	1.05	1.01–1.10	**0.028**
**B. Geometric mean, complete-case analysis**									
Level change	1.01	0.65–1.59	0.96	0.98	0.62–1.57	0.94	0.94	0.55–1.61	0.81
Slope change	1.07	1.03–1.12	**0.00031**	1.10	1.05–1.14	**<0.0001**	1.06	1.01–1.11	**0.016**
**C. Arithmetic mean, negative exposure control**									
Level change	0.97	0.53–1.77	0.92	1.33	0.33–5.41	0.69	0.87	0.58–1.31	0.51
Slope change	1.03	0.97–1.08	0.32	1.09	0.99–1.19	0.072	1.02	0.98–1.06	0.34
**D. Arithmetic mean, excluding eating disorders**									
Level change	0.81	0.34–1.94	0.64	0.79	0.37–1.66	0.53	0.72	0.38–1.35	0.30
Slope change	1.09	1.04–1.14	**0.00025**	1.13	1.07–1.20	**<0.0001**	1.06	1.01–1.12	**0.029**

Footnotes:
ITS = interrupted time series, CAMHS = child and adolescent mental health services, CI = confidence interval.
The combined measure represent the mean durations of both seclusion and mechanical restraint per month. Estimates for intervention effects are expressed as between-group contrasts relative to the reference group (young adult psychiatric inpatients). All confidence intervals and p-values are based on heteroskedasticity- and autocorrelation-consistent (HAC) standard errors. The negative exposure control (C) used a placebo intervention date of July 1, 2018. The exclusion analysis (D) removed all admissions with a main diagnosis of an eating disorder (ICD-10 F50). Panels A and B report ratios of monthly geometric mean durations. Panels C and D report ratios of monthly arithmetic mean durations estimated from log-linear models, with coefficients exponentiated for interpretation. Bolded figures are significant at $p<0.05$.

Table S9. Sensitivity analyses of controlled ITS models of the effect of legal change on sedative medication use in Region Stockholm inpatient CAMHS.

	All sedatives			*Antipsychotics*			*All sedatives excl. melatonin*			*All sedatives excl. antipsychotics*			*All sedatives excl. antipsychotics & melatonin*			*Antipsychotics excl. lithium*		
**Doses per patient-days**																		
	**IRR**	**95% CI**	**p-value**	**IRR**	**95% CI**	**p-value**	**IRR**	**95% CI**	**p-value**	**IRR**	**95% CI**	**p-value**	**IRR**	**95% CI**	**p-value**	**IRR**	**95% CI**	**p-value**
1A. Negative exposure control																		
Level change	0.97	0.85–1.10	0.60	0.91	0.70–1.18	0.46	0.96	0.85–1.10	0.58	1.02	0.83–1.27	0.83	1.04	0.83–0.72	0.72	0.91	0.70–1.19	0.50
Slope change	0.98	0.96–0.99	**0.00079**	0.95	0.93–0.98	**0.00027**	0.97	0.96–0.99	**<0.0001**	1.00	0.98–1.01	0.56	0.99	0.98–0.26	0.26	0.95	0.92–0.97	**<0.0001**
1B. Excluding eating disorder																		
Level change	0.92	0.78–1.09	0.32	0.88	0.66–1.18	0.40	0.85	0.72–1.01	0.061	0.95	0.69–1.29	0.73	0.82	0.58–1.16	0.26	0.94	0.68–1.29	0.69
Slope change	1.00	0.99–1.01	0.66	1.00	0.98–1.02	0.86	1.01	1.00–1.02	0.30	1.00	0.98–1.02	0.81	1.01	0.98–1.04	0.46	0.99	0.97–1.01	0.44
**Proportion of patients**																		
	**OR**	**95% CI**	**p-value**	**OR**	**95% CI**	**p-value**	**OR**	**95% CI**	**p-value**	**OR**	**95% CI**	**p-value**	**OR**	**95% CI**	**p-value**	**OR**	**95% CI**	**p-value**
2A. Negative exposure control																		
Level change	0.99	0.79–1.24	0.94	0.94	0.80–1.11	0.49	1.04	0.85–1.27	0.70	0.98	0.79–1.22	0.85	1.06	0.84–1.33	0.63	0.92	0.78–1.07	0.28
Slope change	0.97	0.96–0.99	**0.00025**	0.97	0.95–0.99	**0.0016**	0.97	0.95–0.98	**<0.0001**	0.98	0.96–0.99	**0.00053**	0.97	0.95–0.99	**0.00083**	0.96	0.94–0.98	**0.00013**
2B. Excluding eating disorder																		
Level change	1.09	0.87–1.35	0.47	1.02	0.75–1.38	0.91	1.01	0.80–1.28	0.92	1.06	0.90–1.26	0.48	0.97	0.81–1.17	0.77	1.03	0.76–1.39	0.87
Slope change	0.98	0.96–1.00	0.059	0.99	0.97–1.01	0.34	0.98	0.96–1.00	**0.019**	0.98	0.96–0.99	**0.0098**	0.97	0.96–0.99	**0.00012**	0.99	0.97–1.01	0.20

Footnotes:
ITS = interrupted time series, IRR = incidence rate ratio. OR = odds ratio. CI = confidence interval.
Estimates for intervention effects are expressed as between-group contrasts relative to the reference group (young adult psychiatric inpatients). All confidence intervals and p-values are based on heteroskedasticity- and autocorrelation-consistent (HAC) standard errors. ATC classification of sedative medications: All sedatives = N05A, N05B, N05C, R06AD01 (alimemazine) and R06AD02 (prometazine); Antipsychotics = N05A; All sedatives except melatonin = N05A, N05B, N05C, R06AD01 and R06AD02, excluding N05CH01 (melatonin); All sedatives except antipsychotics = N05B, N05C, R06AD01 and R06AD02N05; All sedatives except antipsychotics and melatonin = N05B, N05C, R06AD01 and R06AD02N05, excluding N05CH01; Antipsychotics except lithium = N05A excluding N05AN01 (lithium). The negative exposure control analyses (1A and 2A) used a placebo intervention date of July 1, 2018. The exclusion analyses (1B and 2B) removed all admissions with a main diagnosis of an eating disorder (ICD-10 F50). Bolded figures are significant at $p<0.05$.
